# Translation, Cultural Adaptation, and Validation of the Arabic Version of the Type 1 Diabetes Distress Scale (T1-DDS) Among Adults with Type 1 Diabetes in Saudi Arabia

**DOI:** 10.3390/healthcare14010079

**Published:** 2025-12-29

**Authors:** Abdullah M. Alguwaihes, Shuliweeh Alenezi

**Affiliations:** 1 Division of Endocrinology, Department of Internal Medicine, College of Medicine, King Saud University, Riyadh 11437, Saudi Arabia; 2 Department of Psychiatry, College of Medicine, King Saud University, Riyadh 11451, Saudi Arabia; 3 SABIC Psychological Health Research and Applications Chair (SPHRAC), Department of Psychiatry, College of Medicine, King Saud University, Riyadh 11451, Saudi Arabia

**Keywords:** type 1 diabetes, diabetes distress, mental health, insulin pump, depression, anxiety

## Abstract

**Background/Objectives:** Diabetes distress is a significant, often unaddressed, aspect of type 1 diabetes (T1D) management. The Type 1 Diabetes Distress Scale (T1-DDS) is a key assessment tool, but no validated Arabic version exists for Saudi Arabia’s large T1D population. This study aimed to translate, culturally adapt, and validate the Arabic T1-DDS to enable accurate assessment of diabetes-specific distress in Saudi adults with T1D. **Methods:** A cross-sectional study was conducted among Saudi adults with self-reported T1D. The T1-DDS underwent forward-backward translation, expert panel review, and cognitive debriefing. Participants completed an online survey containing the 28-item Arabic T1-DDS, the Patient Health Questionnaire-9 (PHQ-9), and the Generalized Anxiety Disorder-7 (GAD-7). Psychometric evaluation included exploratory and confirmatory factor analysis (EFA, CFA), internal consistency (Cronbach’s α), and convergent validity. A multivariable generalized linear model identified predictors of distress. **Results:** The analysis included 837 participants (73.8% female, mean age 27.22 ± 7.8 years). The Arabic T1-DDS demonstrated excellent reliability (α = 0.934). EFA/CFA supported a robust four-factor structure, confirming model fit (χ^2^/df = 1.313, CFI = 1.000, RMSEA = 0.019). The mean overall distress score was 2.74 (SD = 1.05), with 72.4% reporting moderate-to-high distress. Distress correlated moderately with depression (r = 0.58) and anxiety (r = 0.52). Multivariable analysis identified higher PHQ-9 (RR = 1.178) and GAD-7 scores (RR = 1.063) as significant predictors of elevated distress, while insulin pump use was protective (RR = 0.938). **Conclusions:** The Arabic T1-DDS is a valid and reliable tool for Saudi adults with T1D. Its use revealed a high prevalence of diabetes distress, strongly linked to psychological symptoms and mitigated by insulin pump therapy. Integrating this scale into routine clinical care can facilitate timely psychosocial support, potentially improving patient outcomes.

## 1. Introduction

The Type 1 Diabetes Distress Scale (T1-DDS) is a validated 28-item self-report instrument specifically designed to assess emotional distress among adults with type 1 diabetes (T1D) [1]. It captures various sources of distress unique to living with T1D, including concerns related to diabetes management, interpersonal relationships, healthcare interactions, and emotional burden.

Diabetes distress (DD) and depression are often mistakenly used interchangeably, although both are associated with poor diabetes outcomes; however, they have different diagnostic approaches, implications, and treatment pathways [2]. Diabetes distress is characterized by negative emotional reactions such as frustration, anxiety, and worry specifically related to diabetes management, differing significantly from general psychological conditions like depression and anxiety [3,4]. Elevated diabetes distress has been consistently associated with poorer glycemic control, reduced self-management, and decreased quality of life in individuals with T1D [5,6,7].

Type 1 diabetes is a common chronic autoimmune disease [8]. It has a peak incidence during childhood and adolescence; however, most people living with type 1 diabetes are adults [9]. Globally, we are witnessing an increase in the prevalence and incidence of type 1 diabetes [10]. Currently, there are an estimated 9.5 million people living with type 1 diabetes, representing a 13% increase from the estimate of 8.4 million in 2021 [10]. In 2025, there were an estimated 513,000 new cases of T1D globally [10].

Saudi Arabia is no exception. It is estimated that there are 241,348 people living with type 1 diabetes in the kingdom, among whom 49,118 are less than 20 years old [9]. With an estimated incidence of 31.4 cases per 100,000 youths annually, Saudi Arabia ranks among the top 10 countries worldwide for T1D incidence [10]. These statistics highlight T1D as a significant challenge to the healthcare system in Saudi Arabia, necessitating efforts from health policymakers and providers to improve care, reduce costs, and enhance quality of life. It is well established that uncontrolled diabetes is associated with microvascular and macrovascular complications, which are major contributors to the enormous costs of T1D and reduced life expectancy [10,11,12,13].

Mental well-being is a recognized important component of overall diabetes management, but is unfortunately often overlooked. As emphasized in the American Diabetes Association’s Standards of Care 2025 [14], diabetes management extends well beyond blood glucose control and should include psychosocial care integrated with routine medical care, delivered through a collaborative, person-centered, culturally informed approach [14]. The daily requirements and demands of keeping T1D under control are substantial and represent a real source of ongoing distress for people living with T1D, which has been shown to influence disease control [7,15,16,17]. This has led to the development of various scales to help clinicians measure and assess the psychological and mental aspects of their patients, including the T1-DDS [3,18].

The T1-DDS has been translated and adapted to many cultures [19,20,21,22]. However, an Arabic version culturally adapted to Saudi contexts is lacking. This gap limits the ability of healthcare workers caring for people with T1D (PwT1D) in Saudi Arabia to properly assess sources of distress and implement appropriate interventions. In this study, we aimed to translate, culturally adapt, and validate the Arabic version of the T1-DDS for use among adults with T1D in Saudi Arabia.

## 2. Materials and Methods

### 2.1. Study Design and Participants

This translation, cultural adaptation, and validation study was conducted in Saudi Arabia. Participants were adults aged 18 years or older with a diagnosis of type 1 diabetes (T1D), residing in Saudi Arabia, and fluent in Arabic. Only Saudi nationals with T1D (PwT1D) were included in the analysis to ensure cultural relevance. Exclusion criteria included individuals with type 2 diabetes, gestational diabetes, or other forms of diabetes, as well as those under 18 years or non-Saudi residents.

The study utilized a cross-sectional design with an online questionnaire comprising four sections: (1) demographic and medical data (e.g., age, gender, education level, income, diabetes duration, complications, place of residence, last hemoglobin A1c [HbA1c], diabetes education received, clinical dietitian visits, body mass index [BMI], and exercise habits); (2) the translated Arabic version of the Type 1 Diabetes Distress Scale (T1-DDS); (3) the Arabic-validated Patient Health Questionnaire-9 (PHQ-9) [23]; and (4) the Arabic-validated Generalized Anxiety Disorder-7 (GAD-7) [24].

The questionnaire was hosted on an online platform (Survey Monkey) and disseminated through multiple channels to achieve a broad and diverse reach. The survey link was distributed to PwT1D during clinic visits at diabetes centers, with participants encouraged to share it with their T1D contacts. It was also promoted via T1D support groups on social media platforms, including WhatsApp, Telegram, X (formerly Twitter), and Instagram, as well as through advocacy organizations. Additionally, medical experts with large followers in the T1D community, diabetes educators, and dietitians from various regions were requested to share the link with their patients and professional networks. This strategy, supplemented by snow-bowling recruitment, aimed to maximize participant engagement, geographic diversity, and representation across socioeconomic groups.

The study protocol adhered to international ethical guidelines, including the Declaration of Helsinki. Permission to translate and adapt the T1-DDS was obtained. Ethical approval was granted by the Institutional Review Board of the College of Medicine, King Saud University, Riyadh, Saudi Arabia (Approval Date: 30 May 2021; Reference No. 21/0439/IRB). Data collection was completed between October and December 2021. All participants provided informed consent electronically before completing the questionnaire, and data were anonymized to protect privacy.

### 2.2. Translation and Adaptation Procedure

The translation and cultural adaptation followed established guidelines for cross-cultural validation of patient-reported outcome measures [25,26].

Forward translation was performed independently by a native Arabic speaker proficient in English: a Professor of English Language and Translation from the College of Language Sciences, King Saud University, and another certified translator. The two versions were reconciled in a consensus meeting to resolve discrepancies, ensuring fidelity to the original English T1-DDS while adapting for natural Arabic phrasing. A native Arabic-speaking diabetologist was consulted to address any unresolved differences, particularly regarding diabetes-specific terminology.

Backward translation from Arabic to English, blinded to the original English version, was conducted by a certified professional translation agency unaffiliated with the forward translators. The back-translated version was compared to the original English scale to identify and rectify any semantic shifts.

The preliminary Arabic version was then reviewed by an expert panel comprising two endocrinologists, one diabetologist, one diabetes educator, one PwT1D, and two psychiatrists (initials: MA, IA, EY, SA, SAME, SE). The panel evaluated content validity, cultural appropriateness, clarity, and terminology, making minor adjustments for Saudi cultural nuances (e.g., ensuring items reflected local healthcare practices and social norms). Cognitive debriefing was conducted with a small pilot group of 5 PwT1D to assess comprehension and relevance; no major issues were identified. No items were added or removed, preserving the original 28-item structure.

### 2.3. Measures

#### 2.3.1. Demographic and Medical Data

Self-reported data included sociodemographic variables (age, gender, education level, monthly income, place of residence) and clinical variables (diabetes duration, type of treatment, presence of complications, last HbA1c value, diabetes education clinic visit, clinical dietitian clinic visits, weight, height, and exercise minutes).

#### 2.3.2. Type 1 Diabetes Distress Scale (T1-DDS)

The T1-DDS is a 28-item self-report measure assessing diabetes-specific emotional distress in adults with T1D (1). Items are rated on a 6-point Likert scale from 1 (“not a problem”) to 6 (“a very serious problem”). It comprises seven subscales: powerlessness (5 items), management distress (4 items), hypoglycemia distress (4 items), negative social perceptions (4 items), eating distress (3 items), physician distress (4 items), and friend/family distress (4 items). Total and subscale scores are calculated as means, with higher scores indicating greater distress. The original English version has demonstrated strong reliability (Cronbach’s α > 0.80) and validity.

#### 2.3.3. Patient Health Questionnaire-9 (PHQ-9)

The PHQ-9 is a 9-item scale measuring depressive symptoms over the past two weeks, rated on a 4-point Likert scale from 0 (“not at all”) to 3 (“nearly every day”) [27]. Total scores range from 0 to 27, with cutoffs for mild (5–9), moderate (10–14), moderately severe (15–19), and severe depression (≥20). The Arabic version has shown good reliability (Cronbach’s α = 0.88) and validity in Saudi populations [23].

#### 2.3.4. Generalized Anxiety Disorder-7 (GAD-7)

The GAD-7 is a 7-item scale assessing anxiety symptoms over the past two weeks, rated on a 4-point Likert scale from 0 (“not at all”) to 3 (“nearly every day”) [28]. Total scores range from 0 to 21, with cutoffs for mild (5–9), moderate (10–14), and severe anxiety (≥15). The Arabic version has demonstrated acceptable reliability (Cronbach’s α = 0.79) and validity [24].

The PHQ-9 and GAD-7 were included to evaluate convergent validity, as diabetes distress is conceptually related but distinct from general depression and anxiety. Significant correlations with these measures would support the T1-DDS’s validity while confirming its specificity to diabetes-related burdens.

### 2.4. Statistical Analysis

Continuous variables were described using means and standard deviations (SD) for normally distributed data or medians and interquartile ranges (IQR) for skewed distributions. Categorical variables were summarized using frequencies and percentages. Normality was assessed using the Kolmogorov–Smirnov test and visual inspection of histograms.

Dimensionality of the T1-DDS was evaluated using exploratory factor analysis (EFA) with principal components analysis (PCA), parallel analysis (PA), the minimum average partial (MAP) test, and tests for closeness to unidimensionality. Sampling adequacy was assessed with the Kaiser-Meyer-Olkin (KMO) measure. The number of extractable factors was determined using a combination of the scree plot, PA, MAP test, and eigenvalue criterion (>1).

Structural equation modeling (SEM) was used to examine the presence of a second-order factor in the T1-DDS correlation matrix. Model fit was evaluated using the chi-square test, chi-square/degrees of freedom (CMIN/DF), comparative fit index (CFI), Tucker–Lewis index (TLI), and root mean square error of approximation (RMSEA). Standardized regression coefficients (factor loadings) were reported. Multivariate normality was assessed using AMOS multivariate kurtosis (Mardia’s multivariate kurtosis equivalent) and its critical ratio, with values exceeding conventional thresholds interpreted as evidence of deviation from multivariate normality. Because the indicators included Likert-type/ordinal responses and showed non-normality at the univariate and/or multivariate levels, Maximum Likelihood (ML) estimation with bootstrap correction was applied to obtain robust standard errors and confidence intervals.

Internal consistency reliability was assessed using Cronbach’s alpha and McDonald’s omega. Pearson’s correlation coefficients were calculated to examine bivariate associations between continuous variables.

Multivariable generalized linear models (GLM) with gamma regression were used to identify predictors of overall diabetes distress scores, with associations expressed as exponentiated beta coefficients (risk ratios) and 95% confidence intervals (CI).

All analyses were performed using IBM SPSS (Version 20) Statistics, SPSS AMOS for SEM, and the standalone FACTOR program [29]. Statistical significance was set at *p* < 0.05.

## 3. Results

### 3.1. Participant Characteristics

A total of 837 adults with self-reported type 1 diabetes (T1D) completed all sections of the questionnaire. Sociodemographic and clinical characteristics are summarized in Table 1. The sample was predominantly female (73.8%), with a mean age of 27.22 years (SD = 7.8) and a mean body mass index (BMI) of 24.55 kg/m^2^ (SD = 5.42). Over half of the participants (58.1%) had lived with diabetes for more than 10 years, and (50.2%) resided in the central region of Saudi Arabia.

### 3.2. Internal Consistency and Reliability

The internal consistency of the study-measured questionnaires was assessed using Cronbach’s alpha reliability analysis among a sample of diabetic patients. As shown in Table A1, all three instruments demonstrated excellent to good internal consistency. The 28-item Type 1 Diabetes Distress Scale (T1-DDS) yielded a Cronbach’s alpha of 0.934, indicating excellent internal reliability when measured in an Arabic backtranslated copy. Likewise, the TIDDS questionnaire factor analysis-based subscale scores also showed great internal consistency; each had a Cronbach’s alpha ≥ 0.787, suggesting that people were able to read and understand them equally reliably. The Patient Health Questionnaire (PHQ-9), which assesses depressive symptoms, showed a Cronbach’s alpha of 0.894, reflecting high internal consistency. Similarly, the Generalized Anxiety Disorder questionnaire (GAD-7) demonstrated a Cronbach’s alpha of 0.916, also indicating excellent internal reliability. These findings suggest that all three instruments used in the study are reliable tools for assessing diabetes-related distress, depression, and anxiety symptoms among diabetic patients in the current sample.

### 3.3. Sources of Distress

Mean scores for the seven original T1-DDS subscales and individual items are presented in Table A2. In descending order of mean subscale scores, the primary sources of distress were powerlessness, hypoglycemia distress, eating distress, diabetes management distress, negative social perceptions, family/friends distress, and physician distress.

### 3.4. Exploratory Factor Analysis

An unrestricted exploratory maximum-likelihood factor analysis was conducted on the 28 Arabic T1-DDS items (N = 837) using FACTOR 12.04 [30] (Table A3). The Kaiser-Meyer-Olkin measure indicated excellent sampling adequacy (KMO = 0.937), and Bartlett’s test of sphericity was significant (χ^2^ (378) = 10,106.40, *p* < 0.001).

Parallel analysis, the minimum average partial test, scree plot inspection, and closeness-to-unidimensionality indices (UniCo = 0.961; ECV = 0.838; MIREAL = 0.216) supported a four-factor solution, despite evidence of a strong general distress factor.

Extraction with oblique Promin rotation yielded four factors explaining 55.7% of the common variance (eigenvalues: 10.18, 2.19, 1.80, 1.43). Model fit was excellent: RMSEA = 0.065 (90% CI [0.062, 0.068]), CFI = 0.975, TLI = 0.965, GFI = 0.992, AGFI = 0.989. Inter-factor correlations ranged from 0.48 to 0.60, with factor determinacy coefficients (Φ) of 0.92–0.95 and ORION reliabilities of 0.846–0.908.

The four factors were labeled as: (1) Provider-Related Distress (loadings 0.76–0.93); (2) Regimen & Self-Care Distress (loadings 0.47–0.80); (3) Hypoglycemia Fear (loadings 0.70–0.77); and (4) Social & Interpersonal Distress (loadings 0.39–0.85). This structure differed from the original seven-factor model [1], with conceptual merging of related domains (e.g., management, eating, and hypoglycemia distress into Regimen & Self-Care Distress).

### 3.5. Confirmatory Factor Analysis and Model Fit

**Figure 1:**
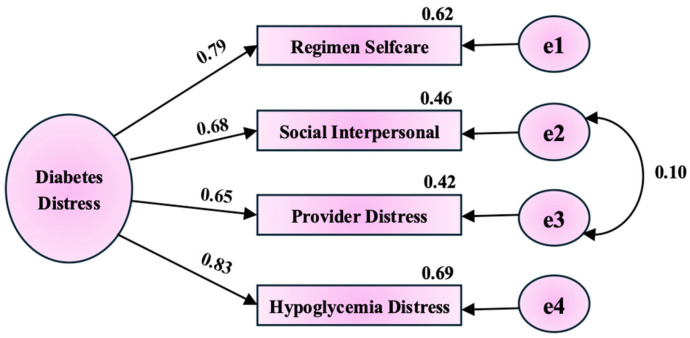
Structural Equation Modelling based path model.

Confirmatory factor analysis (CFA) was performed to evaluate a second-order model with the Maximum Likelihood estimation with 500 bootstrapping were applied where the four factors loaded onto an overall diabetes distress construct (Figure 1, Table A3 and Table A4). Model fit was excellent: χ^2^ (1) = 1.313, *p* = 0.252 (χ^2^/df = 1.313); RMR = 0.005; GFI = 0.999; AGFI = 0.992; CFI = 1.000; TLI = 0.999; RMSEA = 0.019 (90% CI [0.000, 0.097], PCLOSE = 0.623). Parsimony-adjusted indices were acceptable (PNFI = 0.167, PCFI = 0.167). The Standardized loadings for the four subscales onto the overall distress factor were ≥ 0.648. The interpersonal and provider-related distress subscales were allowed to correlate (r = 0.18). Reliability for the overall factor was strong: McDonald’s omega = 0.792, greatest lower bound = 0.842, Cronbach’s α = 0.789, factor determinacy index = 0.898, and EAP marginal reliability = 0.806.

### 3.6. Prevalence, Predictors and Concurrent Validity

The descriptive analysis in (Table A5) provides a clear overview of patients’ overall perception of diabetes distress. When categorized, 38.1% of patients reported high distress (T1-DDS ≥ 3 points), 34.3% reported moderate distress, and 27.6% reported low distress, indicating that nearly two-thirds of patients experience at least moderate levels of distress related to their diabetes.

Bivariate correlations (Table 2). The bivariate correlations presented in Table 2 demonstrate strong internal coherence of the T1-Diabetes Distress Scale (T1-DDS), as evidenced by the robust positive associations between the overall T1-DDS score and its four subscales. Overall diabetic distress was strongly correlated with Social and Interpersonal Distress (r = 0.81, *p* < 0.01), Hypoglycemia Fear/Distress (r = 0.89, *p* < 0.01), Regimen and Self-care Distress (r = 0.81, *p* < 0.01), and Healthcare Provider Distress (r = 0.73, *p* < 0.01), supporting the internal structure and construct validity of the T1-DDS.

To further evaluate convergent and discriminant validity, associations between diabetic distress and general psychological symptoms were examined using the PHQ-9 and GAD-7 scales. As hypothesized, overall diabetic distress showed moderate, statistically significant correlations with depressive symptoms (PHQ-9; r = 0.58, *p* < 0.01) and anxiety symptoms (GAD-7; r = 0.52, *p* < 0.01). These findings support convergent validity, indicating that higher diabetes-specific distress is associated with greater emotional symptom burden. The T1-DDS subscales showed consistent, moderate positive correlations with both depressive (PHQ-9) and anxiety (GAD-7) symptoms. Hypoglycemia Fear/Distress demonstrated the strongest associations with PHQ-9 (r = 0.55, *p* < 0.01) and GAD-7 (r = 0.50, *p* < 0.01), followed by Regimen and Self-care Distress, Social and Interpersonal Distress, and Healthcare Provider Distress. These findings indicate that diabetes-specific distress—particularly hypoglycemia-related concerns—is meaningfully associated with broader emotional symptoms while remaining a distinct construct, supporting the convergent and discriminant validity of the T1-DDS subscales.

At the same time, the magnitude of these correlations remained below thresholds typically indicative of construct redundancy, supporting discriminant validity and confirming that diabetic distress represents a distinct construct rather than a proxy for depression or anxiety. At the subscale level, Hypoglycemia Fear/Distress exhibited the strongest associations with both PHQ-9 (r = 0.55, *p* < 0.01) and GAD-7 scores (r = 0.50, *p* < 0.01), highlighting hypoglycemia-related concerns as a particularly salient psychological stressor in diabetes management.

In contrast, behavioral and anthropometric variables, including daily exercise minutes and body mass index (BMI), were not significantly correlated with diabetic distress or psychological symptom measures. Age showed weak but statistically significant negative correlations with hypoglycemia fear, anxiety, and depressive symptoms, suggesting a slightly higher psychological burden among younger patients. Age was positively associated with BMI, consistent with established age-related trends.

Collectively, these findings underscore the psychological specificity of diabetes-related distress and its meaningful—but non-redundant—relationship with broader affective symptoms, thereby reinforcing the psychometric validity of the T1-DDS in this sample.

To investigate variation in perceived diabetes-related distress, we fitted generalized linear multivariable models with a Gamma distribution (log link) to patients’ mean T1-DDS score. Candidate predictors were chosen a priori based on clinical relevance and a targeted literature review; competing specifications were compared iteratively to yield a parsimonious final model. The final model (Table 3) identified several independent predictors of higher distress: anxiety (per one-unit increase in GAD-7, RR = 1.063, 95% CI [1.027, 1.100], *p* = 0.001) and depressive symptoms (per one-unit increase in PHQ-9, RR = 1.178, 95% CI [1.137, 1.220], *p* < 0.001).

Significantly, insulin pump (CSII) use was associated with lower distress relative to other insulin regimens (e.g., MDI) (RR = 0.938, 95% CI [0.890, 0.989], *p* = 0.018), suggesting a potential protective effect of advanced insulin delivery methods. Sociodemographic variables (sex, age, BMI, income, education) were not significant after adjustment (all *p* > 0.05). Overall, the model highlights the primacy of psychological factors and treatment modality—particularly the beneficial association of insulin pump therapy—over demographics in shaping diabetes-related distress, supporting integrated psychological and educational interventions within routine diabetes care.

## 4. Discussion

This study successfully translated, culturally adapted, and validated the Arabic version of the Type 1 Diabetes Distress Scale (T1-DDS) for use among Saudi adults with type 1 diabetes (T1D). Key findings include excellent internal consistency (Cronbach’s α = 0.934), a four-factor structure differing from the original seven-factor model, a mean overall distress score of 2.74 (SD = 1.05), and a prevalence of moderate-to-high distress in 72.4% of participants. Distress was predominantly driven by regimen and self-care burdens, hypoglycemia fear, and to a lesser extent, provider and interpersonal issues. Concurrent validity was supported by moderate correlations with depression (r = 0.58) and anxiety (r = 0.52), while multivariable analysis identified higher anxiety and depression scores as strong predictors of elevated distress, with insulin pump use offering a protective effect.

### 4.1. Comparison with Previous Studies on Prevalence

The prevalence of moderate-to-high diabetes distress in our sample (72.4%) is notably higher than reported in several prior studies from Saudi Arabia, which ranged from 35% to 53%. For instance, Aljohani et al. [31] found 53.4% prevalence using the Diabetes Distress Scale-17 (DDS-17) among T1D adults, with a mean score of 3.13. Similarly, AlOzairi et al. [32] reported 36.3% in a predominantly type 1 diabetes cohort, and Soliman et al. [17] noted 51% medium-to-high distress across type 1diabetes and type 2 diabetes patients using the DDS-17. However, direct comparisons are limited due to methodological differences: these studies used the general DDS-17 or the Problem Areas in Diabetes (PAID) scale, both which are general scales and more suited to type 2 diabetes and may not capture T1D-specific stressors like insulin dependence and hypoglycemia risks. The pathophysiology and lived experiences of T1D differ substantially from type 2 diabetes, potentially underestimating distress in T1D populations when using non-specific tools. Our use of the T1-DDS, designed explicitly for T1D, likely provides a more sensitive measure, explaining the higher prevalence observed. This aligns with a recent national survey in Saudi Arabia reporting high rates of perceived depressive (50.3% mild-to-severe) and anxiety symptoms (47.7% mild-to-severe) among T1D adults using PHQ-9 and GAD-7, with females showing greater anxiety vulnerability [33]. Internationally, T1-DDS studies report moderate-to-high distress in 30–50% of adults, though cultural and sampling variations exist [18,20].

### 4.2. Cultural Background of Stress and Diabetes in the Saudi Population

Diabetes distress in Saudi Arabia is influenced by unique cultural, social, and healthcare factors. In Arab societies, including Saudi Arabia, strong family interdependence may amplify interpersonal distress, as evidenced by items related to family worry over hypoglycemia scoring relatively high in our sample. Stigma around chronic illnesses, rooted in cultural norms emphasizing self-reliance and concealment of vulnerabilities, could exacerbate negative social perceptions and emotional burdens [30]. Additionally, religious practices like fasting during Ramadan pose unique challenges for T1D management, potentially heightening regimen-related distress—a factor not fully captured in Western scales but relevant here [17]. Our findings of powerlessness and hypoglycemia fear as top stressors resonate with regional studies linking distress to limited access to advanced technologies (e.g., insulin pumps) and education in conservative healthcare settings [32,33]. Broader Arab cultural contexts, such as gender roles (with our female-predominant sample possibly reflecting higher emotional reporting among women, consistent with greater anxiety in females reported elsewhere), and socioeconomic disparities, further contextualize these results [13,33]. These elements underscore the need for culturally tailored interventions, such as integrating family counseling and faith-based support, to mitigate distress in Saudi T1D populations.

### 4.3. Validity and Applicability

The Arabic T1-DDS demonstrated robust psychometric properties, with excellent reliability and convergent validity, supporting its use in Saudi clinical settings. However, exploratory factor analysis revealed a four-factor structure (Provider-Related Distress, Regimen & Self-Care Distress, Hypoglycemia Fear, and Social & Interpersonal Distress), contrasting the original seven-factor model [1]. This parsimonious structure, confirmed by CFA with excellent fit indices, may reflect cultural integration of domains: for example, management, eating, and hypoglycemia concerns merged due to overlapping daily experiences in Saudi contexts, where family and provider roles are closely intertwined. Similar adaptations have been noted in other non-Western validations; for instance, the Brazilian version emphasized cultural phrasing but retained seven factors [22], while the Greek adaptation showed good internal structure without specifying factor changes [19]. The Danish and Norwegian versions largely confirmed the original structure but with minor adjustments for local contexts [20,21]. Our findings suggest the Arabic T1-DDS is applicable for screening in Arabic-speaking populations, offering a shorter, culturally sensitive alternative that maintains theoretical integrity while optimizing measurement stability.

### 4.4. Associations, Predictors, and Future Directions

Bivariate and multivariable analyses highlighted strong links between diabetes distress and psychological factors, with anxiety and depression as key predictors—consistent with global evidence that distress amplifies mental health burdens in T1D [7,16]. The protective role of insulin pumps against elevated distress (RR = 0.938) is supported by recent Saudi data associating pump use with lower odds of severe depression (OR = 0.38) and anxiety (OR = 0.42), particularly among females and those with better glycemic control [33]. This underscores the value of advanced technologies in alleviating management-related stressors, warranting policy efforts to address barriers to accessing new technology. Sociodemographic variables were non-significant, suggesting that distress surpasses demographics in this cohort.

The study’s strength includes a large sample from different regions of Saudi Arabia and the use of Arabic validated tools such as PHQ-9 and GAD-7 to confirm validity of our work. However, limitations include reliance on self-reported data and snowball sampling, which may introduce bias toward more engaged or distressed individuals, limiting generalizability. The cross-sectional design precludes causality, and the female-skewed sample may not fully represent male experiences. Future research should employ longitudinal designs, diverse sampling (e.g., rural areas), and test–retest reliability. Comparative studies with other Arabic-speaking countries could refine the scale further, and interventions targeting identified predictors (e.g., pump education, anxiety screening) should be evaluated.

## 5. Conclusions

This study successfully developed and validated the first Arabic version of the T1-DDS specifically adapted for the Saudi cultural context. The Arabic T1-DDS demonstrated robust psychometric properties, including excellent reliability and a stable, culturally relevant four-factor structure, confirming its validity for use with Saudi adults with type 1 diabetes. The application of this scale in a large national sample revealed a critically high prevalence of diabetes-related distress, which was predominantly driven by psychological comorbidity. A key and actionable finding is the significant protective association between insulin pump therapy and lower distress levels.

Therefore, the integration of the Arabic T1-DDS into routine clinical practice is strongly recommended to facilitate the systematic screening and identification of diabetes distress. This can pave the way for timely, person-centered interventions, which should include integrated psychological support and efforts to improve access to advanced diabetes technologies. Future longitudinal and interventional studies are needed to confirm these cross-sectional associations and to develop effective strategies for mitigating distress in this population.

## Figures and Tables

**Table 1:** Sociodemographic characteristics. n = 837.

		Frequency	Percentage
**Sex**	Female	618	73.8
	Male	219	26.2
**Age**	(Mean Age in Years ± SD)	27.22 (7.8)	
**Body Mass Index (BMI), mean (SD)**		24.55 (5.42)	
**last HbA1c results?**	≤7%	313	37.4
	7.1–8%	256	30.6
	8.1–9%	122	14.6
	>9%	120	14.3
	I do not know	26	3.1
**Educational Level**			
	High school or less	212	25.3
	University	557	66.6
	Post-graduate study	68	8.1
**Diabetes Duration**	<1 years	53	6.3
	1–5 years	152	18.2
	6–10 years	146	17.4
	>10 years	486	58.1
**Diabetes Management**	Insulin injections	655	78.3
	Insulin pump	182	21.7
**Daily exercise (minutes), median (IQR)**		8.57 (14.28)	
**Residence region**	Central region	420	50.2
	Western region	221	26.4
	Eastern region	87	10.4
	North region	47	5.6
	Southern region	62	7.4

**Table 2:** Bivariate correlations between patients’ measured perceptions.

Parameter	T1-DDS	SD	HYPO	RD	PRO	GAD7	PHQ9	EXER	BMI
Overall Type 1 Diabetes Distress (T1-DDS) score.	1.000								
Social & Interpersonal (SD) Distress	0.806 **								
Hypoglycemia (HYPO) Fear/Distress	0.886 **	0.549 **							
Diabetes Regimen and (RD) self-care Distress	0.811 **	0.547 **	0.652 **						
Healthcare Provider (PRO) Distress	0.731 **	0.494 **	0.550 **	0.492 **					
Generalized Anxiety Disorder (GAD7) score	0.515 **	0.422 **	0.502 **	0.386 **	0.309 **				
Patient Health (PHQ9) score	0.584 **	0.470 **	0.548 **	0.436 **	0.411 **	0.788 **			
Daily exercise minutes (EXER)	−0.012	−0.005	−0.034	−0.025	0.048	−0.038	−0.058		
Body Mass Index (BMI) Score	0.000	0.008	0.028	−0.039	−0.025	0.027	0.030	0.043	
Age (years)	−0.052	−0.065	−0.091 **	0.056	−0.025	−0.088 *	−0.083 *	0.027	0.227 **

** Correlation is significant at the 0.01 level (2-tailed). * Correlation is significant at the 0.05 level (2-tailed).

**Table 3:** Multivariable Generalized Linear regression with Gamma for patients’ mean perceived type 1 diabetes distress (T1-DDS) score.

Parameter	Multivariable Adjusted Risk Rate	95% CI for RR		*p*-Value
		Lower	Upper	
(Intercept)	2.472	2.179	2.804	<0.001
Sex = Male	1.039	0.989	1.091	0.131
Age (years)	1.000	0.996	1.003	0.758
Body Mass Index (BMI) Score	0.999	0.995	1.003	0.679
Household Monthly Income (SR)	0.991	0.976	1.005	0.214
Educational level	1.015	0.988	1.044	0.280
Used type of Insulin Management = Insulin Pump	0.938	0.890	0.989	0.018
Zscore: Generalized Anxiety Disorder (GAD7) score	1.063	1.027	1.100	0.001
Zscore: Patient Health (PHQ9) score	1.178	1.137	1.220	<0.001

Dependent Variable: Overall Diabetes Distress (T1-DDS) score. Maximum likelihood estimates with Gamma Regression.

## Data Availability

The data that support the findings of this study are available upon request from the corresponding author. The data are not publicly available due to privacy or ethical restrictions.

## Appendix A

**Table A1:** Internal consistency and reliability analysis.

	Number of Items	Cronbach’s Alpha
The T1-DDS is a 28-item self-report instrument.	28	0.934
Factor analysis-based TIDDS subscales reliability analysis		
Social & Interpersonal Distress	8	0.861
Hypoglycemia Fear/Distress	10	0.900
Regimen and Self-Care Distress	6	0.787
Healthcare Provider Distress	4	0.871
The Patient Health (PHQ-9) questionnaire	9	0.894
The Generalized Anxiety Disorder (GAD7) questionnaire	7	0.916

**Table A2:** Descriptive analysis of patients’ perceived disease (T1-DDS) distress indicators.

	Mean	Standard Deviation
**Management-related distress**		
“MD1. Feeling that I am not as skilled at managing diabetes as I should be.”	2.84	1.56
“MD8. Feeling that I am not taking as much insulin as I should.”	2.17	1.55
“MD12. Feeling that I don’t check my blood glucose level as often as I probably should.”	2.70	1.78
“MD28. Feeling that I don’t give my diabetes as much attention as I probably should.”	2.71	1.78
**Eating Distress**		
ED2. Feeling that I don’t eat as carefully as I probably should.	3.20	1.65
ED16. Feeling that thoughts about food and eating control my life.	2.96	1.79
ED23. Feeling that my eating is out of control.	3.23	1.75
**Negative Social Perceptions**		
NSP4. Feeling that people treat me differently when they find out I have diabetes.	2.62	1.74
NSP10. Feeling like I have to hide my diabetes from other people.	2.30	1.75
NSP19. Feeling concerned that diabetes may make me less attractive to employers.	2.78	1.85
NSP24. Feeling that people will think less of me if they knew I had diabetes.	2.20	1.68
**Hypoglycemia distress**		
HD3. Feeling that I don’t notice the warning signs of hypoglycemia as well as I used to.	2.68	1.76
HD15. Feeling frightened that I could have a serious hypoglycemic event when I’m asleep.	3.26	1.83
HD22. Feeling frightened that I could have a serious hypoglycemic event while driving.	2.84	1.80
HD27. Feeling that I can’t ever be safe from the possibility of a serious hypoglycemic event.	2.65	1.68
**Powerlessness**		
PW5. Feeling discouraged when I see high blood glucose numbers that I can’t explain.	3.89	1.73
PW9. Feeling that there is too much diabetes equipment and stuff I must always have with me.	2.55	1.70
PW13. Feeling worried that I will develop serious long-term complications, no matter how hard I try.	3.59	1.93
PW21. Feeling that I’ve got to be perfect with my diabetes management.	2.95	1.81
PW25. Feeling that no matter how hard I try with my diabetes, it will never be good enough.	2.98	1.75
**Friends/Family Distress**		
FFD6. Feeling that my family and friends make a bigger deal out of diabetes than they should.	2.64	1.80
FFD11. Feeling that my friends and family worry more about hypoglycemia than I want them to.	2.74	1.73
FFD17. Feeling that my friends or family treat me as if I were more fragile or sicker than I really am.	2.24	1.66
FFD20. Feeling that my friends or family act like “diabetes police” (bother me too much).	2.18	1.64
**Physicians Distress**		
PD7. Feeling that I can’t tell my diabetes doctor what is really on my mind.	2.72	1.85
PD14. Feeling that I don’t get the help I really need from my diabetes doctor about managing diabetes.	2.63	1.90
PD18. Feeling that my diabetes doctor doesn’t really understand what it’s like to have diabetes.	2.34	1.79
PD26. Feeling that my diabetes doctor doesn’t know enough about diabetes and diabetes care.	2.22	1.78

**Table A3:** Promax-Rotated Pattern Matrix Loadings for the Arabic Version of the T1-DDS (n = 837).

Item	Factor 1 (Provider-Related Distress)	Factor 2 (Regimen & Self-Care Distress)	Factor 3 (Hypoglycemia Fear)	Factor 4 (Social & Interpersonal Distress)
Feeling that I am not as skilled at managing diabetes as I should be.	0.022	0.030	0.753	−0.055
Feeling that I don’t eat as carefully as I probably should.	0.028	0.018	0.723	−0.060
Feeling that I don’t notice the warning signs of hypoglycemia as well as I used to.	0.158	0.295	0.138	−0.053
Feeling that people treat me differently when they find out I have diabetes.	−0.064	−0.052	−0.049	0.744
Feeling discouraged when I see high blood glucose numbers that I can’t explain.	−0.117	0.398	0.376	0.025
Feeling that my family and friends make a bigger deal out of diabetes than they should.	−0.077	−0.190	−0.004	0.865
Feeling that I can’t tell my diabetes doctor what is really on my mind.	0.445	0.017	0.254	0.096
Feeling that I am not taking as much insulin as I should.	0.241	−0.024	0.497	−0.032
Feeling that there is too much diabetes equipment and stuff I must always have with me.	−0.013	0.293	0.209	0.106
Feeling like I have to hide my diabetes from other people.	0.007	0.100	0.051	0.430
Feeling that my friends and family worry more about hypoglycemia than I want them to.	−0.088	0.074	−0.057	0.624
Feeling that I don’t check my blood glucose level as often as I probably should.	0.125	−0.167	0.700	0.028
Feeling worried that I will develop serious long-term complications, no matter how hard I try.	−0.036	0.234	0.460	0.070
Feeling that I don’t get the help I really need from my diabetes doctor about managing diabetes.	0.761	−0.008	0.114	−0.031
Feeling frightened that I could have a serious hypoglycemic event when I’m asleep.	0.032	0.772	−0.053	−0.046
Feeling that thoughts about food and eating control my life.	−0.056	0.280	0.449	0.113
Feeling that my friends or family treat me as if I were more fragile or sicker than I really am.	0.067	0.009	−0.041	0.785
Feeling that my diabetes doctor doesn’t know enough about diabetes and diabetes care.	0.887	0.050	−0.102	0.036
Feeling concerned that diabetes may make me less attractive to employers.	0.101	0.222	0.098	0.364
Feeling that my friends or family act like ‘diabetes police’ (bother me too much).	0.091	−0.065	−0.015	0.772
Feeling that I’ve got to be perfect with my diabetes management.	−0.088	0.226	0.619	−0.053
Feeling frightened that I could have a serious hypoglycemic event while driving.	0.035	0.720	−0.086	−0.020
Feeling that my eating is out of control.	−0.064	0.064	0.807	−0.069
Feeling that people will think less of me if they knew I had diabetes.	0.024	0.178	0.028	0.512
Feeling that no matter how hard I try with my diabetes, it will never be good enough.	−0.022	0.298	0.540	0.006
Feeling that my diabetes doctor doesn’t understand what it’s like to have diabetes.	0.943	−0.010	−0.125	−0.030
Feeling that I can’t ever be safe from the possibility of a serious hypoglycemic event.	0.083	0.719	0.014	−0.005
Feeling that I don’t give my diabetes as much attention as I probably should.	0.119	−0.147	0.735	−0.010

Note. Extraction method: Maximum -Likelihood. Rotation method: Promax with Kaiser normalization. Loadings ≥ 0.30 are shown. No Heywood cases observed; rotation converged in 6 iterations.

**Table A4:** Confirmatory Factor Analysis (CFA) standardized regression coefficients for the four Factor T1-DDS.

Domains	Standardized Regression Coefficient
Regimen Distress	0.787
Interpersonal Distress	0.676
Provider Distress	0.648
Hypoglycemia Distress	0.829

**Table A5:** Descriptive analysis for patients’ overall perception of diabetes distress.

Type 1 Diabetes Distress (T1-DDS) Level	No	Percentage
Low Distress (T1-DDS < 2 points)	231	27.6
Moderate Distress (T1-DDS = 2–2.9 points)	287	34.3
High distress (T1-DDS ≥ 3 points)	319	38.1
